# The Osteocyte Stimulated by Wnt Agonist SKL2001 Is a Safe Osteogenic Niche Improving Bioactivities in a Polycaprolactone and Cell Integrated 3D Module

**DOI:** 10.3390/cells11050831

**Published:** 2022-02-28

**Authors:** Yangxi Liu, Xiaojie Ruan, Jun Li, Bo Wang, Jie Chen, Xiaofang Wang, Pengtao Wang, Xiaolin Tu

**Affiliations:** Laboratory of Skeletal Development and Regeneration, Institute of Life Sciences, Chongqing Medical University, Chongqing 400016, China; 2020111818@stu.cqmu.edu.cn (Y.L.); ruanxiaojie@ezedy.cn (X.R.); lijun@cqifdc.org.cn (J.L.); 2019111169@stu.cqmu.edu.cn (B.W.); 2019010260@stu.cqmu.edu.cn (J.C.); 2019111156@stu.cqmu.edu.cn (X.W.); 2019111164@stu.cqmu.edu.cn (P.W.)

**Keywords:** SOOME, osteocyte, Wnt signaling, MLO-Y4, ST2, osteoblast differentiation, PCL and cell integrated 3D printing

## Abstract

Finding and constructing an osteogenic microenvironment similar to natural bone tissue has always been a frontier topic in orthopedics. We found that osteocytes are targeting cells controlling bone anabolism produced by PTH (JBMR 2017, PMID: 27704638), and osteocytes with activated Wnt signaling orchestrate bone formation and resorption (PNAS 2015, PMID: 25605937). However, methods for taking advantage of the leading role of osteocytes in bone regeneration remain unexplored. Herein, we found that the osteocytes with SKL2001-activated Wnt signaling could be an osteogenic microenvironment (SOOME) which upregulates the expression of bone transcription factor *Runx2* and *Bglap* and promotes the differentiation of bone marrow stromal cell ST2 into osteoblasts. Interestingly, 60 μM SKL2001 treatment of osteocytic MLO-Y4 for 24 h maintained Wnt signaling activation for three days after removal, which was sufficient to induce osteoblast differentiation. Triptonide, a Wnt inhibitor, could eliminate this differentiation. Moreover, on day 5, the Wnt signaling naturally decreased to the level of the control group, indicating that this method of Wnt-signaling induction is safe to use. We quickly verified in vivo function of SOOME to a good proximation in 3D bioprinted modules composed of reciprocally printed polycaprolactone bundles (for support) and cell bundles (for bioactivity). In the cell bundles, SOOME stably supported the growth and development of ST2 cells, the 7-day survival rate was as high as 91.6%, and proliferation ability increased linearly. Similarly, SOOME greatly promoted ST2 differentiation and mineralization for 28 days. In addition, SOOME upregulated the expression of angiopoietin 1, promoted endothelial cell migration and angiogenesis, and increased node number and total length of tubes and branches. Finally, we found that the function of SOOME could be realized through the paracrine pathway. This study reveals that osteocytes with Wnt signaling activated by SKL2001 are a safe osteogenic microenvironment. Both SOOME itself and its cell-free culture supernatant can improve bioactivity for osteoblast differentiation, with composite scaffolds especially bearing application value.

## 1. Introduction

There are several sources of bone defects, including infection, tumor resection, complex trauma, revisional surgery, etc. [1]. More than 1.6 million bone grafts are needed in the United States every year, which creates a great social and economic burden [2]. The global orthopedic implant market grows at 4.7% yearly and will reach USD 60 billion by 2025 [3]; however, the current products still face two challenges: anatomic feature mismatching and lack of bioactivity, resulting in instability, looseness and need for renovation, which is a headache for surgeons. The 3D printing of hard materials has emerged as a particularly attractive candidate for reconstruction of personalized bone implants due to its capacity to use computed tomography (CT)-derived images or computer-aided design (CAD) models [4,5,6], but more attention needs to be given to improving material bioactivity in order to regenerate the patient’s own bone.

For bone regeneration, doctors make great efforts to use autogenous and allergenic bone for transplantation. The use of autogenous bone remains the so-called “gold standard” of transplantation; however, it is limited by long operation time, insufficient sources and high infection rates [7,8,9], not to mention allergenic bone that causes immune rejection [7]. With the development of bone tissue engineering, 3D printing technology has removed the shortage of traditional bone grafts by allowing on-demand printing of the required structure and shape of bone materials, and has also created an opportunity for the development of functional bone grafts [10,11]. However, the predominant materials adopted for bone implant fabrication through 3D printing are metals, ceramics and thermoplastic polymers, which are all melted under high temperature, eliminating any chance to incorporate cells during fabrication. Furthermore, post-treatment strategies to include cells face issues such as uneven cell distribution. Bioprinting is a promising option to construct bioactive bone implants with cells included by reciprocally alternating plotting of cell-laden hydrogel and thermoplastic polymers [12,13,14]. However, low bioactivity is still a bottleneck to overcome.

In order to further improve the bioactivity of bone grafts, biological components, primarily growth factors such as BMP, VEGF, etc., have been successfully combined with various biomaterials to fabricate bone implants [15,16]. Although they potentially promote skeletal tissue regeneration, there are corresponding side effects, e.g., ectopic ossification, osteolysis and swelling in the case of BMP [17]. The ultimate goal of 3D printing is to fabricate artificial tissues or organs similar to natural ones in structure and function. At present, although printed organ-like tissues, including skin, liver, bone, cartilage, cardiovascular and neuron structures, have been quickly adopted in high-throughput drug screening and discovery, they are still in the early stage of research for the creation of functional organs [18].

The biological functions of the cellular microenvironment play a vital role in maintaining the physiological homeostasis of skeletal tissue, as well as bone repair and regeneration [19]. There are three main types of cells involved in bone regeneration: osteoblasts and osteoclasts, which maintain structural balance, and osteocytes, which respond to environmental, mechanical or chemical signal stimulation [20]. Recently, researchers have gained a more comprehensive understanding of the function of osteocytes: in addition to acting as mechanical sensing cells and sending signals to other recipient cells, such as osteoblasts and osteoclasts, they also act as important endocrine cells to secrete many biological factors that have strong effects on osteoblasts and osteoclasts, controlling the bone microenvironment [21,22,23]. For example, osteocytes secrete sclerostin, an effective inhibitor of Wnt signaling, to negatively regulate osteoblast differentiation and survival [24,25,26]. 

Wnt signaling regulates the developmental and postnatal processes through classical/non-classical pathways, including cell proliferation, differentiation, polarization and migration [27]. β-catenin is the central transducer involved in the classical pathway, wherein Wnt ligand binds to its receptor Frizzled and related receptor low density lipoprotein (LRP) 5/6 to inactivate GSK3β to stabilize β-catenin in the cytoplasm. Then the stabilized β-catenin enters the nucleus and interacts with Wnt-signaling-factor Tcf/Lef family members, activating the transcription of Wnt target genes. 

We have found that activation of Wnt signaling in osteocytes mediates bone formation and resorption, a physiological function for bone anabolism [28,29]. We found such a function of osteocytes not only in in vivo study by gene manipulation, but also in ex vivo study with separated genuine osteocytes with Wnt activation. In this study, we aimed to generate an osteogenic microenvironment by defining the function of the osteocytes that are stimulated by Wnt agonist SKL2001 (S24) for 24 h, resulting in activation of Wnt signaling in these osteocytes for 3 days upon removal of S24.

Wnt agonists have been widely explored for the treatment of orthopedic-related diseases [30,31]; small molecular drugs with good stability are particularly useful candidates [32]. S24 can effectively activate Wnt signaling without inhibiting the activity of GSK3β, which reduces the risk of adverse effects on cells or tissues [31]. Based on our knowledge, this drug has not been used for bone regeneration and repair. We postulate that S24-treated osteocytes are a safe osteogenic microenvironment (SOOME), as could be evidenced in SOOME 3D modules biofabricated by our newly established hard material and cell-integrated 3D bioprinting system.

We show herein that SOOME could keep Wnt signaling elevated for 3 days, specifically promoting osteoblast differentiation and mineralization in bone marrow stromal cells. The three-dimensional culture mimicking an in vivo experiment found that SOOME stably supports cell growth on hard materials with a 7-day survival rate as high as 91.6% and significantly increased proliferation capacity, and SOOME supports osteoblast differentiation and mineralization for up to 28 days, which solves the problems of cell survival and growth on hard materials, a long-standing question in bone tissue engineering. Interestingly, SOOME promotes angiogenesis of endothelial cells as well. This study reveals that SOOME is a safe osteogenic microenvironment for bone tissue regeneration and repair.

## 2. Materials and Methods

### 2.1. Reagents and Cells

Chemicals: Wnt signaling agonist SKL2001 (S24) and Wnt signaling inhibitor triptonide were purchased from MedChemExpress (Shanghai, China), Lyophilized Gelatin Methacryloyl (GelMA) and photo-cross-linking agent lithium phenyl-2, 4, 6-trimethylbenzoylphosphinate (LAP) from Sunp biotech (Beijing, China), polycaprolactone (PCL) from Sigma (St. Louis, MO, USA), Trizol from Invitrogen (Thermo Fisher Scientific, Carlsbad, CA, USA), and matrigel from Corning (Costar; Corning, NY, USA). Alizarin red S staining was from Solarbao Biotechnology (Beijing, China).

Assay kits: BCIP/NBT alkaline phosphatase (AP) color-rendering kit, live/dead viability assay kit, alkaline phosphatase activity assay kit, DAPI staining solution and crystal violet staining solution were obtained from Beyotime Biotechnology (Shanghai, China), AG RNAex Pro Reagent and reverse transcription reaction kits from Accurate Biology (Changsha, Hunan, China), and CCK-8 assay kit from MedChemExpress (Shanghai, China).

Antibodies: polyclonal anti-β-catenin antibody was supplied by Wanlei (Shenyang, Liaoning, China), and FITC labeled goat anti-rabbit IgG (H + L) was obtained from Beyotime Biotechnology (Shanghai, China). 

Reagents of cell culture and cell line: α-MEM, penicillin and streptomycin were purchased from Gibco (Grand Island, NY, USA), fetal bovine serum (FBS) from Biological Industries (Kibbutz Beit Haemek, Israel), Wnt3a-expressing cells, control L cells and human umbilical vein endothelial cells (HUVECs) from the American Type Culture Collection (ATCC) (Manassas, VA, USA). MLO-Y4 was kindly provided by Professor Lynda Bonewald, Indian University School of Medicine. ST2 cells were obtained from Dr. Steve Teitelbaum, Washington University.

### 2.2. Cell Culture

Cell culture of murine cell line ST2, murine osteocyte-like immortalized cell line MLO-Y4, Wnt3a-expressing cells and control L cells were performed as previously report [33,34,35]. These cells were cultured in 90% α-MEM, 10% FBS supplemented with 50 U/mL penicillin and 50 μg/mL streptomycin. HUVECs were cultured in Dulbecco’s Modified Eagle Medium (DMEM) supplemented with 10% FBS, 100 U/mL penicillin and 100 μg/ mL streptomycin. Half of the medium was changed once every two days. DMSO and S24 treated MLO-Y4 and ST2 cells were co-cultured in each well of a 24-well plate at a density of 0.5 × 10^4^ and 2 × 10^4^ cells, respectively, for main research.

### 2.3. Activation and Inhibition of Wnt Signaling in Osteocytic MLO-Y4 

S24 was used to activate the Wnt signal pathway of MLO-Y4, and we defined the S24-treated MLO-Y4 as the SOOME. Meanwhile, MLO-Y4 treated by DMSO acted as the control. Triptonide was used to inhibit the Wnt signal pathway by binding to the C-terminal transactivation domain of β-catenin to silence β-catenin action [34]. For activation of Wnt signaling in osteocytic MLO-Y4, MLO-Y4 cells were split in each well of a 24-well plate at a density of 3 × 10^4^ cells and treated with S24 at concentrations of 0, 10, 30, 40, 60 or 100 μM or at the concentration as indicated for 24 h. As for measuring the specificity of the role of Wnt signaling in MLO-Y4 on osteoblast differentiation, MLO-Y4 cells were treated with S24 at 60 μM for 24 h, followed with triptonide treatment (10 nM) for another 24 h to inhibit Wnt signaling in MLO-Y4. 

### 2.4. PCL and Cell Integrated 3D Printing

A PCL and cell-integrated 3D (PCI3D) module was biofabricated through a dual-head printer—one head prints PCL bundle for support and the other prints cell-laden hydrogel bundle for bone formation. These two bundles were reciprocally printed on one layer. For each additional layer, the printed PCL beams and cell beams were perpendicular to the ones of previous layer.

Lyophilized GelMA was fully dissolved in α-MEM medium to prepare a 20% (*w*/*v*) GelMA solution containing 0.5% (*w*/*v*) lithium phenyl-2, 4, 6-trimethylbenzoylphosphinate (LAP) photo-initiator. Then 2 × 10^5^ MLO-Y4 cells and 8 × 10^5^ ST2 cells were mixed and suspended in 0.5 mL α-MEM. The cell suspension was mixed with an equal volume of GelMA solution for 3D bioprinting. 

The cell-loaded GelMA solution was placed into a syringe and pre-cooled at 4 °C for 5 min, and then the syringe was put in the cell-printing nozzle for extrusion by air pressure. Polycaprolactone (PCL) was put in a hard material nozzle and melted at 95 °C. Then the melted PCL was plotted at 400 μm diameter with an interval at 1100 μm at a printing speed of 2 mm/s. The PCL was printed as the frame structure of the scaffold, and then the cell-loaded GelMA solution was plotted between the PCL strips at 300 μm diameter with a 500 μm interval between cell bundles at a printing speed of 5 mm/s. 

After printing, GelMA hydrogel was crosslinked under blue light at 405 nm for 30 s. Each layer was printed on the previous one, perpendicularly, to form a 0°/90° strut structure until 4 layers were deposited. The printed PCI3D modules were placed in cell growth medium in a 6-well plate and cultured at 37 °C and 5% CO_2_ for subsequent experiments. Group 1 was the control group, DMSO-treated MLO-Y4 cells co-cultured with ST2 cells. Group 2 was the SOOME group, S24 treated MLO-Y4 cells co-cultured with ST2. Each group was repeated in triplicate.

### 2.5. Cell Viability Assay

Cell viability in PCI3D modules was quantitated by a live/dead viability assay kit. After one, four, seven day of cell culture, the cells were washed by PBS and incubated in the staining mixture, which was prepared by adding 1 μL Calcein AM (1000×) and 1 μL PI (1000×) in 1 mL of assay buffer. Calcein AM was used to dye the living cells, and PI was used to dye the dead cells [36]. The plates were incubated in the incubator for 0.5 h, and the samples were imaged immediately using an inverted fluorescence microscope [36] (Leica, Wetzlar, Germany). Cell viability was quantified using the ImagJ software (64-bit, v1.46).

### 2.6. Cell Proliferative Activity

Cell counting kit-8 was used per manufacturer’s instructions to evaluate the proliferation activity of cells in the functional modules at days 1, 4 and 7 after initiation of culture. PCI3D modules were washed with PBS, cut into four pieces and placed in a 96-well plate. A total of 10 μL CCK-8 in 100 μL PBS was added to each well, and then the plate was incubated for 2 h [37]. Subsequently, the supernatant was pipetted into a new 96-well plate and absorbance at 450 nm was measured by using a microplate reader [38].

### 2.7. RNA Extraction and Gene Expression Analysis

Total RNA extraction and quantitative PCR (qPCR) were performed as reported [39]. Briefly, total RNA was extracted from the cells treated with Trizol as previously described. The cDNA was synthesized by using a high-capacity cDNA reverse transcription kit (TaKaRa, Shiga, Japan) and used as the template for qPCR with primer sets (Table 1). Relative mRNA expression levels were normalized to the housekeeping gene glyceraldehyde-3-phosphate dehydrogenase (Gapdh) by using the Δ Ct method [29]. 

### 2.8. Alkaline Phosphatase Staining

After 3 days of cell culture, the cells were washed with PBS (Sorlabio, Beijng, China) and fixed in 3.7% formaldehyde (Chongqing Chuandong Chemical Group, Chongqing, China) for 5 min at room temperature. ALP staining was performed on the cells for 30 min by using the BCIP/NBT alkaline phosphatase color development kit [40]. It should be noted the ST2 and MLO-Y4 cells in the 3D module are stained after 7 and 14 days and the staining time is extended to four hours. The staining results were recorded by a digital camera.

### 2.9. AP Biochemical Activity Assay

AP biochemical activity assay was performed as previously described [35]. After 0.3 mL 50 mM Tris/HCl (pH 7.4) was added to each well, the cells were removed from the plate by scratching. The whole process was performed on ice. After 3-min centrifugation at 12,000 rpm, the supernatant was used for assay with the AP detection kit step-by-step per procedures.

### 2.10. Detection of Translocation of β-Catenin into the Nucleus

MLO-Y4 cells were cultured on the 24-well plate treated with S24 or DMSO. After 24 h, they were washed with PBS, fixed with 4% paraformaldehyde, permeabilized for 30min with PBS containing 0.25% Triton X-100 (PBS-T), and blocked with 1% BSA in PBS. Immunostaining was performed using the rabbit polyclonal anti-mouse β-catenin antibody (1:50) and FITC labeled goat anti-rabbit IgG (H + L) secondary antibody (1:500) as described [14]. Samples were washed three times in PBS, one of which included a 5-min incubation in DAPI (1:5000), and washed again [41]. Then images were acquired using a fluorescence microscope.

### 2.11. Mineralization Assay (Alizarin Red S Staining)

PCI3D modules were first cultured in the growth medium for seven days. The mineral nodule formation was induced in the osteogenic medium containing 0.1 mM dexamethasone, 10 mM β-glycerophosphate disodium salt and 50 μg/mL L-ascorbic acid for another 21 days. Matrix mineralization was analyzed by alizarin red S staining [35]. The modules were stained with 0.4% alizarin red S for 30 min and photographed under a microscope. Then the modules were extensively washed with PBS at room temperature, and the samples were destained in 10% cetylpyridinium chloride for 1 h. The washing solution was measured for absorbance at 562 nm for quantitation of mineralization status [42].

### 2.12. In Vitro HUVEC Tube Formation Assay

A concentration of 0.3 × 10^5^ cells/cm^2^ MLO-Y4 and 1.2 × 10^5^ cells/cm^2^ HUVECs treated with DMSO or S24 were mixed and seeded onto Matrigel (200 μL) in pre-coated 24-well plates and cultured for six hours in an incubator as reported [43]. Capillary-like structures appeared and were counted under a microscope. The network of vascular tubes formed from HUVEC cells was quantified with ImageJ. 

### 2.13. Conditioned Medium Preparation

Conditioned medium was prepared as previously reported [44]. Briefly, MLO-Y4 cells were treated with S24 for 24 h, then the cells were washed in PBS and continuously cultured in an S24-free medium for another 24 h. At the end of treatment, the conditioned medium was collected and stored at −80 °C until use. In parallel, the control conditioned medium was collected from MLO-Y4 treated with DMSO. Wnt3a conditioned medium and its control L medium were collected as previously reported [45]. Wnt3a conditioned medium was used as a positive control and L medium as a negative control.

### 2.14. Cell Migration Assay

Transwell migration assay was performed as previously [43]. A chamber containing a polycarbonate filter with a pore size of 8.0 μm (Costar; Corning, NY, USA) was used for passage of migrated cells. HUVECs were serum-starved for 12 h. The chambers were filled with 800 μL of 10% FBS αMEM with 200 μL conditional medium. Approximately 1 × 10^5^ HUVECs were added to the upper chamber. Cells were incubated at 37 °C for 48 h to allow cell migration through the membrane. The lower chambers were incubated with crystal violet staining solution to stain the migrated cells. The migrated cells were imaged using a microscope and were quantified with ImageJ.

### 2.15. Statistical Analysis

Statistical analysis was performed by GraphPad Prism 8.0.1 software. Each experiment was repeated three times independently. The data were presented as mean ± SD. The normal distribution of data was confirmed by using normality and lognormality tests. One-way ANOVA was used to analyze the differences between multiple groups and two-way ANOVA was used to analyze the differences between groups that have been split on two independent variables. Meanwhile, Brown—Forsythe and Welch ANOVA tests were used for correction analysis. A *t*-test was performed between two comparable groups. Nonparametric Kruskal-Wallis test was used to compare medians instead when data distributions are not normal. Significant difference was considered as *p* < 0.05.

## 3. Results

### 3.1. Effects of S24 on Activation of Wnt/β-Catenin Signaling in Osteocytic MLO-Y4

**Figure 1:**
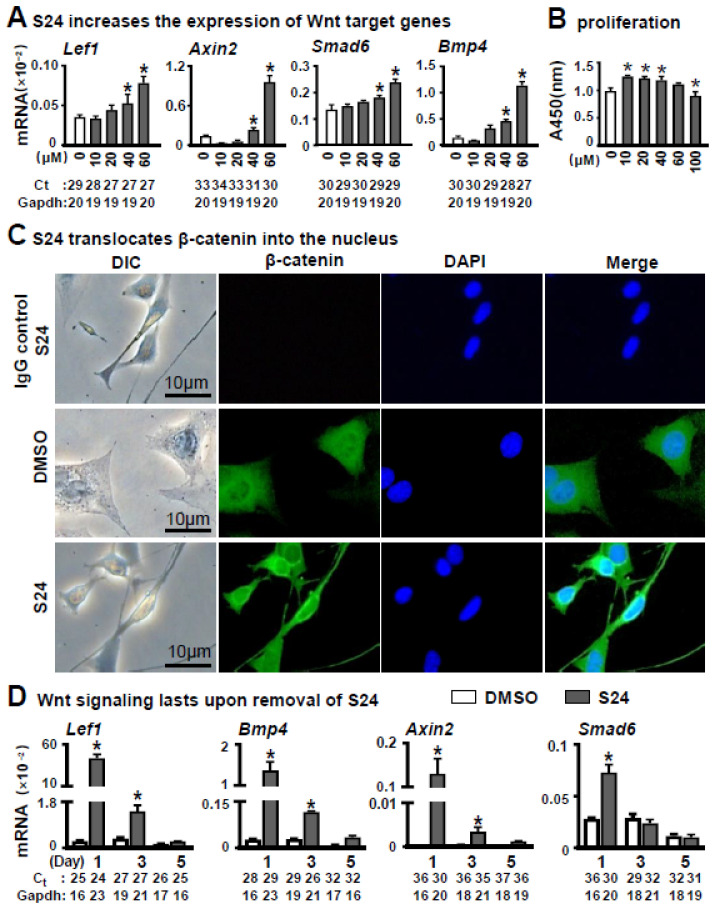
Effects of S24 on canonical Wnt signaling on osteocytic MLO-Y4 cells. (**A**) S24 increased the expression of Wnt target genes detected by qPCR in MLO-Y4 cells treated with DMSO (control) or S24 (10, 20, 40 or 60 µM) for 24 h. Level of mRNA versus housekeeping gene *Gapdh*. The * indicates *p* < 0.05 versus DMSO group by one-way ANOVA; n = 3. (**B**) Effect of S24 on cell proliferative activity measured by CCK-8 assay. Results were represented as relative OD450 values, which were calculated by normalizing the OD450 reading to the mean value of the control. S24 at 60 μM had no significant effect on MLOY4’s proliferation activity. (**C**) Immunofluorescence detected β-catenin in MLO-Y4 cells treated with 60 µM S24 or DMSO. Anti-β-catenin antibodies were used to detect β-catenin levels in the nuclei and DAPI was used to stain the nucleus. Scale bar = 10 µm. (**D**) The maintaining time of Wnt signaling in MLO-Y4 cells after removal of S24. The expression of Wnt target genes was detected in these cells by qPCR on days 1, 3 and 5 after removal of S24. Ct, cycle number detected by qPCR machine for a relative amount of target genes.

To determine the effect of Wnt signaling-activated osteocytes on osteoblast differentiation of stromal cells, we selected Wnt activator S24 for activating the Wnt pathway in osteocytes. MLO-Y4 was treated with S24 at a concentration of 0, 10, 30, 40 or 60 μM for 24 h. Then qPCR confirmed the upregulation of Wnt target genes, such as *Lef1*, *Axin2*, *Bmp4* and *Smad6*, by S24 in a dose-dependent manner (Figure 1A). 

CCK-8 assay demonstrated that S24 at 60 μM had less effect on proliferation activity of MLO-Y4 cells within 24 h (Figure 1B). Whereas proliferative activity was increased upon S24 treatment at concentrations 10, 20 and 40 μM, and decreased at 100 μM as compared to the non-treatment control. Therefore, we used 60 μM S24 in the study unless indicated otherwise. 

Meanwhile, 60 μM S24-stimulated MLO-Y4 for 24 h also displayed a maximum enhancement in osteoblast differentiation of ST2 cells as detected by AP staining and the expression of osteoblast marker genes compared to 0, 10 and 30 μM S24-treated MLO-Y4 cells. These data showed a dose-dependent way for S24 to induce osteoblast differentiation (Figure S1A,B).

To further determine whether S24 activated the Wnt signaling by the canonical pathway, we investigated the level of intranuclear β-catenin by immunofluorescence assay using anti-β-catenin antibody. As expected, treatment with 60 μM S24 resulted in an increase in β-catenin level in the nuclei (Figure 1C), indicating that S24 promoted the translocation of β-catenin to the nucleus.

As we were going to test the effect of Wnt signaling-activated osteocytes on osteoblast differentiation, we measured how long Wnt signaling could last in the S24-treated MLO-Y4. Wnt target genes were detected in these cells by qPCR on days 1, 3 and 5 after S24 treatment. Although the expression of Wnt target genes gradually decreased, mRNA levels of *Lef1* and *Bmp4* were significantly higher in S24-treated cells than the ones in DMSO control on days 1 and 3, but had comparable expression with the control on day 5 (Figure 1D). Thus, one day of treatment with 60 μM S24 could activate Wnt signaling over at least three days. 

### 3.2. Effects of S24-Stimulated Osteocytes (SOOME) on Osteoblast Differentiation

**Figure 2:**
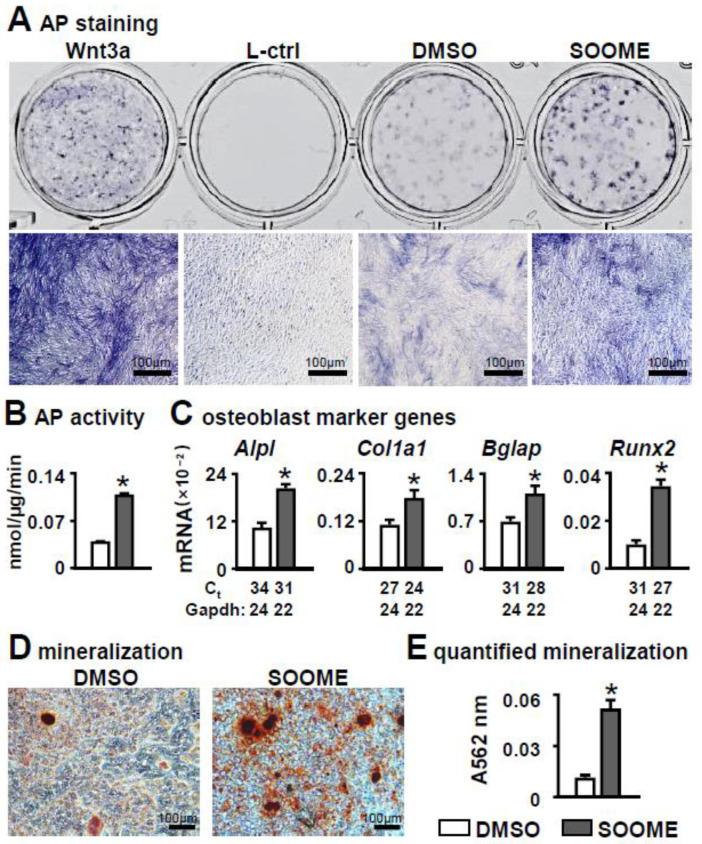
Effects of Wnt signaling-activated osteocytes on osteogenic differentiation in stromal cells. MLO-Y4 was treated with 60 µm S24 for 24 h, then co-cultured with ST2 cells for 3 days, and osteogenic differentiation was evaluated by AP staining (**A**). Wnt3a conditioned medium and its L control medium were used as positive and negative controls, respectively. Lower images are enlargements of upper images; scale bar = 100 µm. (**B**) AP biochemical activity assay. (**C**) Expression of osteoblast marker genes as measured by qPCR. The * symbol indicates *p* < 0.05 by Student *t*-test; n = 3. (**D,E**) Mineralization assay of ST2 cells co-cultured with SOOME in growth medium for 3 days and in osteogenic medium for another 14 days. The osteogenic medium was changed every three days. The mineralized bone nodules were photographed after alizarin red S staining, followed by quantification assay. The * symbol indicates *p* < 0.05 versus DMSO group by *t*-test; n = 3. Scale bar = 100 µm. Ct, cycle number detected by qPCR machine for a relative amount of target genes.

After co-cultured with mouse stromal cell line ST2 for three days, the S24-stimulated MLO-Y4 cells significantly promoted osteoblast differentiation of ST2 cells. This is evidenced the co-culture with enhanced AP activities in the assays of AP staining and its biochemical activity (Figure 2A,B), and with increased expression of osteoblast marker genes in qPCR detection (Figure 2C) as compared with the control counterparts. All these parameters were almost detected from the ST2 cells due to their much lower levels in MLO-Y4 cells (with or without S24 treatment) than the co-culture of MLO-Y4 and ST2 cells (Figure S4A–C). S24 also significantly promoted mineralization, as indicated by the bigger and denser mineral nodules formed in the S24-treated MLO-Y4 group as tested by alizarin red S staining (Figure 2D), in which S24-treated MLO-Y4 promoted a 2.4-fold increase in calcium deposition compared to the DMSO control (Figure 2E). These data suggest that the S24-stimulated osteocyte is an osteogenic microenvironment (SOOME, hereafter) promoting osteoblast differentiation.

To investigate the specificity of SOOME in the enhancement of osteoblast differentiation, we tested whether a non-bone cell line NIH 3T3 fibroblast induced osteoblast differentiation in ST2 after being similarly treated with S24 for 24 h. However, the results were negative (Figure S2A), and vice versa—SOOME did not induce NIH 3T3 fibroblasts to osteoblasts (Figure S2B).

### 3.3. Specificity of Wnt/β-Catenin Signaling in SOOME on Osteoblast Differentiation

**Figure 3:**
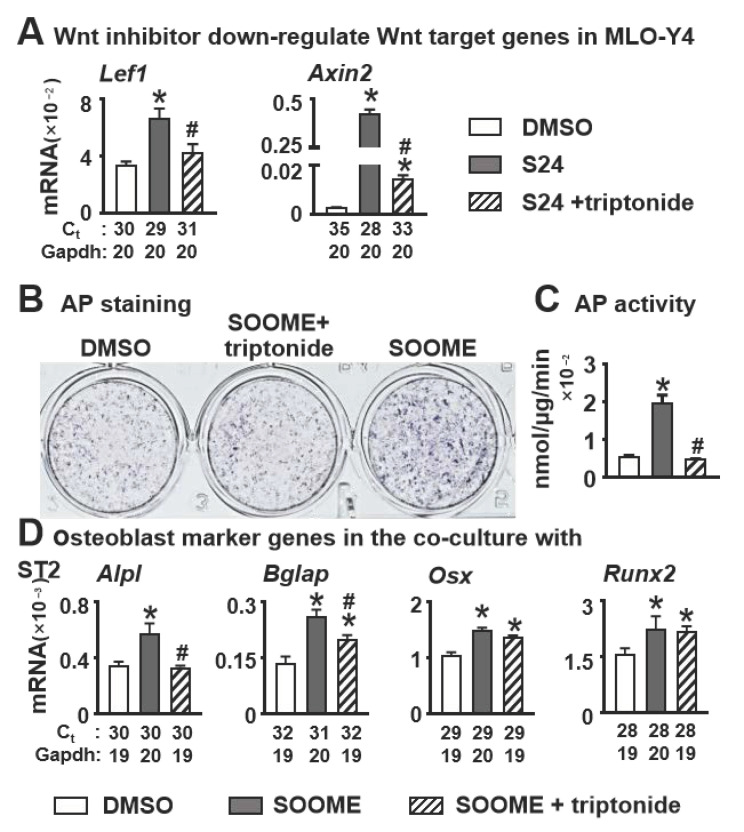
Effects of inhibition of Wnt/β-catenin signaling in the SOOME on osteoblast differentiation. The SOOME was treated with or without Wnt inhibitor triptonide (10 nM) for 24 h. (**A**) qPCR detected the expression of Wnt target genes in the treated MLO-Y4 cells as indicated. Then the treated SOOME was co-cultured with ST2 cells for 3 days. Osteoblast differentiation was evaluated by (**B**) AP staining, (**C**) AP biochemical activity assay and (**D**) qPCR for the expression of osteoblast marker genes in co-culture system. The * symbol indicates *p* < 0.05 versus DMSO control and ^#^ indicates *p* < 0.05 versus SOOME by one-way ANOVA, Brown—Forsythe and Welch ANOVA tests and Kruskal—Wallis test; n = 3. Ct, cycle number detected by qPCR machine for a relative amount of target genes.

To confirm whether S24-activated canonical Wnt signaling in osteocytes is required for osteoblast differentiation of stromal cells, we used the Wnt inhibitor triptonide in the assay. Triptonide binds to the C-terminal transactivation domain of β-catenin, silencing β-catenin action. The qPCR results demonstrated that triptonide at 10 nM insufficiently downregulated the expression of Wnt target genes *Lef1* and *Axin2* (Figure 3A), which were upregulated by SOOME. Subsequently, the enhanced osteoblast differentiation by SOOME was decreased to the control level by triptonide (Figure 3B–D), indicating that SOOME-activated Wnt signaling in osteocytes is responsible for its function on osteoblast differentiation.

As we further examined the osteogenic factor of SOOME, we found that the expression of the PGE2 receptor *EP4* was upregulated both in SOOME and the co-culture of SOOME and ST2 cells (Figure S3D,E). Meanwhile, Wnt inhibitor triptonide decreased the expression of *EP4*, suggesting that PGE2 may be involved, which needs to be further studied. In addition, triptonide reduces the expression of Wnt target genes in co-culture, which may reduce osteoblast differentiation below that of the control group (Figure S3A–C), indicating SOOME may have generated Wnt ligand(s) and upregulated Wnt target genes (Figure S3A–C).

### 3.4. Effects of SOOME on Cell Viability and Proliferation in PCI3D Modules 

**Figure 4:**
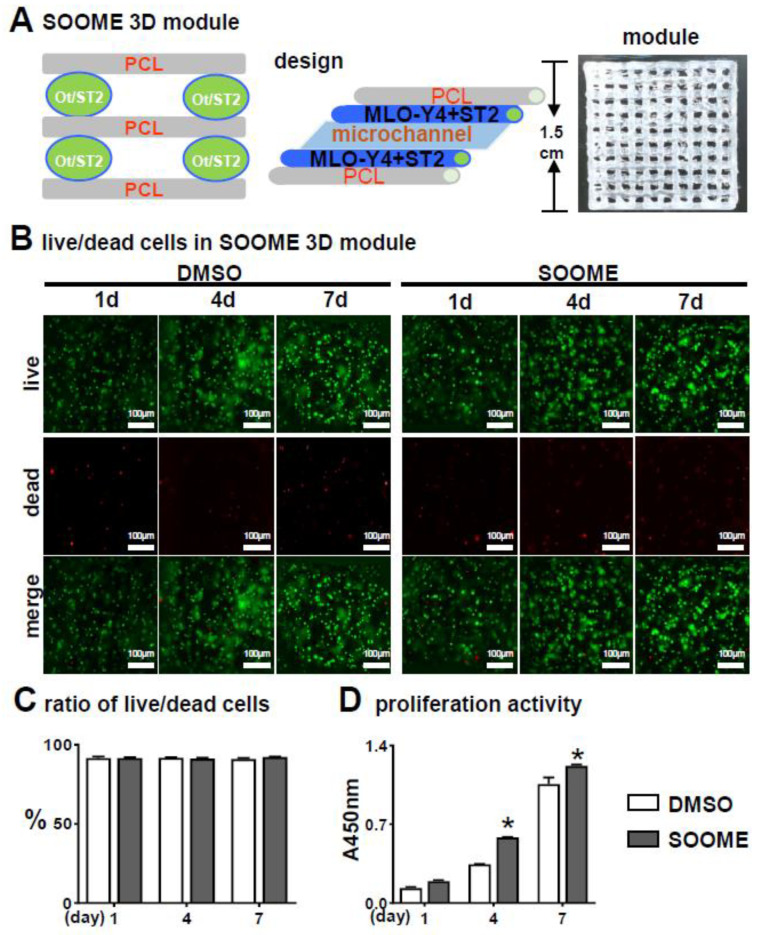
Effects of the SOOME in PCI3D modules on cell survival and growth. (**A**) A schematic PCI3D system design and its printed module image (right) reciprocally printed with PCL beams and cell-laden hydrogel beams. (**B**) Live/dead cell assay to detect the cells’ survival and proliferation at predetermined time points (1, 4 and 7 days after in vitro culture). Cells displayed calcein-AM positive (green) for live cells or EthD-1 negative (red) for dead cells. Scale bar = 100 µm. (**C**) The rate of live/dead cells and (**D**) proliferation activity was analyzed by imaging and CCK8 assay kit. The * symbol indicates *p* < 0.05 versus DMSO control by Two-way ANOVA; n = 3.

To make a good proximation to the real in vivo microenvironment, we examined the biological activities of SOOME in a 3D bioprinted module [46]. The SOOME 3D module was fabricated by our established PCL and cell-integrated 3D printing (PCI3D) system. PCL bundles were extruded from the heating cartridge with a screw propeller to support the SOOME 3D modules. The cells were loaded in hydrogel with S24-activated MLO-Y4 and ST2 at ratio of 1:4. This module possessed stable support and a tunnel-connection structure with a suitable aperture size for the transportation of nutrition and metabolic materials (Figure 4A).

The PCI3D modules with S24-treated MLO-Y4 and ST2 were cultured for one day and followed by Calcein AM and PI staining for the live/dead cell assay. Figure 4B–C illustrates the high cell viability between these functional modules of MLO-Y4-Wnt (91.7%) and MLO-Y4 control (91.4%).

CCK8 assay on proliferation activity for seven consecutive days showed that all the cells in the PCI3D modules had linear increases in cell proliferation capacity (Figure 4D). The SOOME group displayed higher proliferation activity compared to the control groups at day 4 and 7. These results suggest that the PCI3D module provided a favorable environment for cell survive and proliferation, and that SOOME promoted stromal cell proliferation.

### 3.5. Effects of SOOME on Osteogenesis and Mineralization in PCI3D Modules 

**Figure 5:**
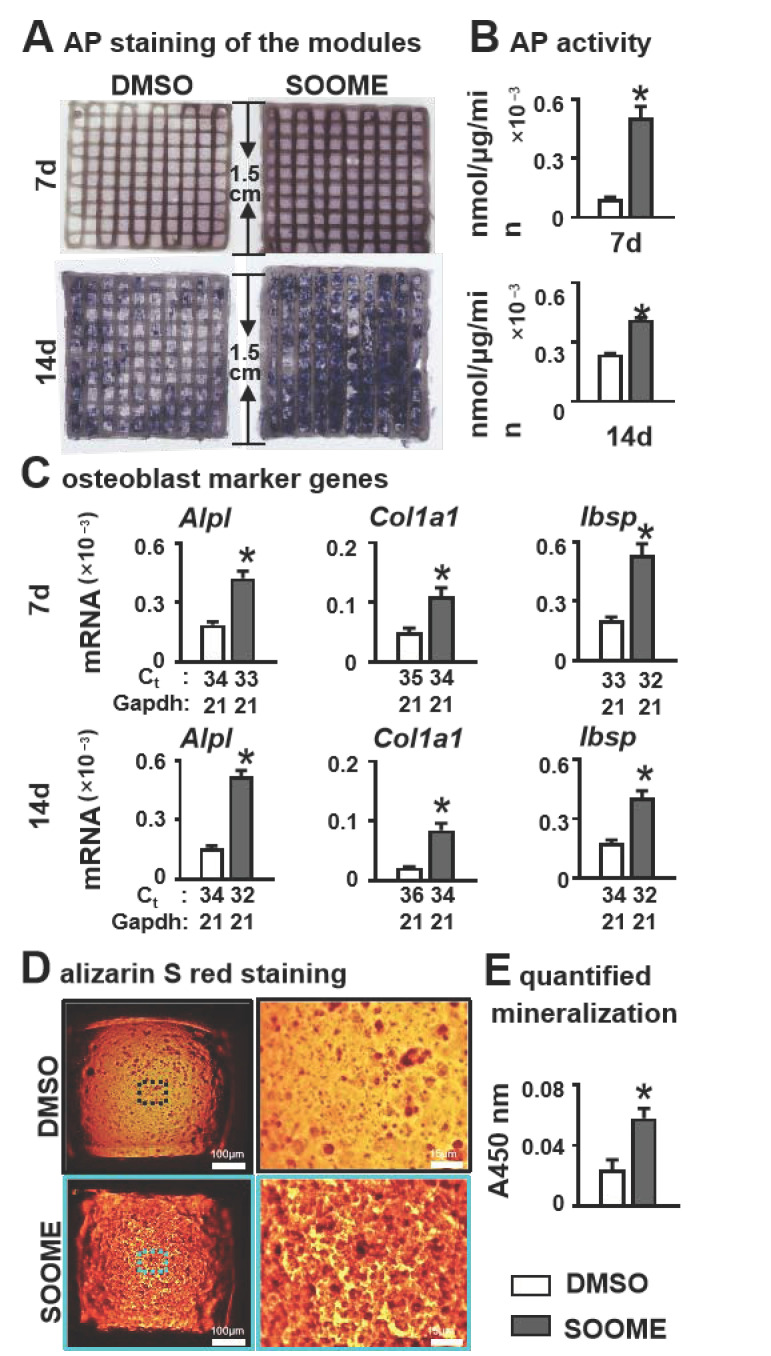
Effects of SOOME in PCI3D modules on osteoblast differentiation and mineralization of stromal cells. Wnt-activated MLO-Y4 promoted osteogenic differentiation of stromal cells in PCI3D modules with SOOME and ST2 cells. After being cultured for 7 and 14 days, the cells were subjected to (**A**) AP staining; (**B**) AP activity assays; and (**C**) qPCR for the expression of osteoblast marker genes. (**D**,**E**) Increased mineralization of the PCI3D modules. Modules were cultured in full growth medium for 7 days, followed by culture in the osteogenic medium supplemented with ascorbic acid, β-glycerophosphate disodium and dexamethasone. Alizarin red S staining revealed denser formed bone nodules in SOOME 3D modules. The * symbol indicates *p* < 0.05 versus DMSO control; n = 3. Scale bar = 200 µm. Ct, cycle number detected by qPCR machine for a relative amount of target genes.

Before we started the experiment, we detected whether the 3D system induces osteoblast differentiation in ST2 cells. We found no effects of the hydrogel with LAP on osteoblast differentiation in ST2 cells (Figure S5). We further determined the effects of SOOME on osteogenesis of stromal cells by mimicking the real in vivo microenvironment to a good proximation [46]. The SOOME 3D modules were cultured for 7 and 14 days and then subjected to osteoblast differentiation assays. SOOME significantly increased osteoblast differentiation as measured by AP staining and biochemical activity assays in the PCI3D modules as compared with DMSO control modules (Figure 5A,B). This was further confirmed by qPCR quantification, which showed that the SOOME group displayed a significantly increased gene expression of osteoblast markers, such as *Alpl*, *Col1a1* and *Ibsp*, when compared to control group (Figure 5C).

To evaluate the effects of SOOME on bone nodule formation, PCI3D modules were cultured in full growth medium for 7 days, followed by cultivation in an osteogenic medium. Alizarin red S staining revealed denser mineral nodules in SOOME modules, with a 2.3-fold increase in calcium deposition compared to DMSO control modules (Figure 5D,E). Our results further confirmed the positive effect of SOOME on osteogenesis of ST2 cells.

### 3.6. Effect of SOOME on Angiogenesis

**Figure 6:**
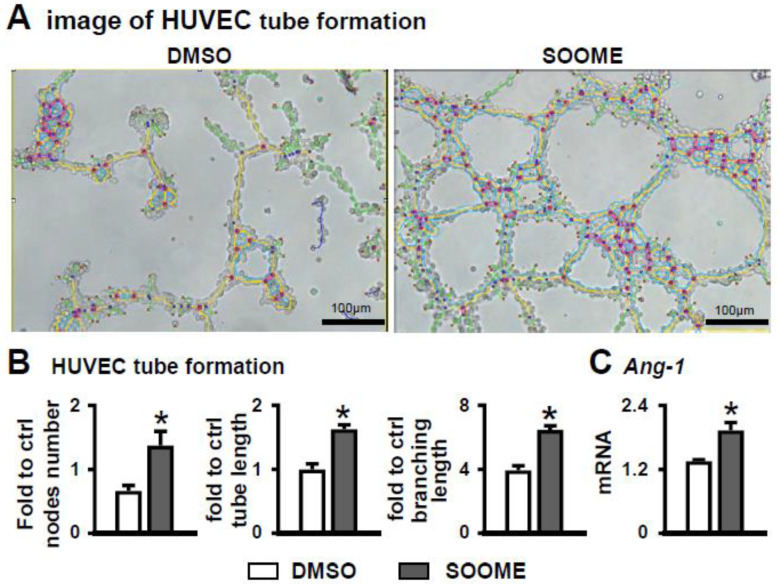
Effects of SOOME on angiogenesis of HUVECs. HUVECs co-cultured with SOOME at a cell ratio of 1:4 (osteocyte:HUVEC) on Matrigel in 24-well plate for 6 h until formed blood-vessel-like structures. Imaging (**A**) and quantification of node formation (**B**) in the co-culture of SOOME and HUVECs. (**C**) qPCR detected the expression of Angiopoietin-1 in the co-culture. The * symbol indicates *p* < 0.05 versus DMSO control; n = 3. Scale bar = 100 µm. Green = branches; orange = master segments; blue sky = meshes; red surrounded by blue = nodes; Ct, cycle number detected by qPCR machine for a relative amount of target genes.

To explore whether SOOME induced angiogenesis, we measured the tube formation of co-cultured HUVECs with SOOME. SOOME increased vascular tubule formation compared to the DMSO control group (Figure 6A). The numbers of nodes, total length of tubules and branching were increased by 2.1-, 1.6- and 1.6- fold, respectively, in the SOOME group compared to the DMSO group (Figure 6B). We also found that the SOOME upregulated the expression of angiogenic Ang1 genes in HUVEC cells compared to the DMSO control (Figure 6C). These results suggest that SOOME enhanced angiogenesis.

### 3.7. Effect of Conditioned Medium of SOOME on Osteoblast Differentiation and Angiogenesis

**Figure 7:**
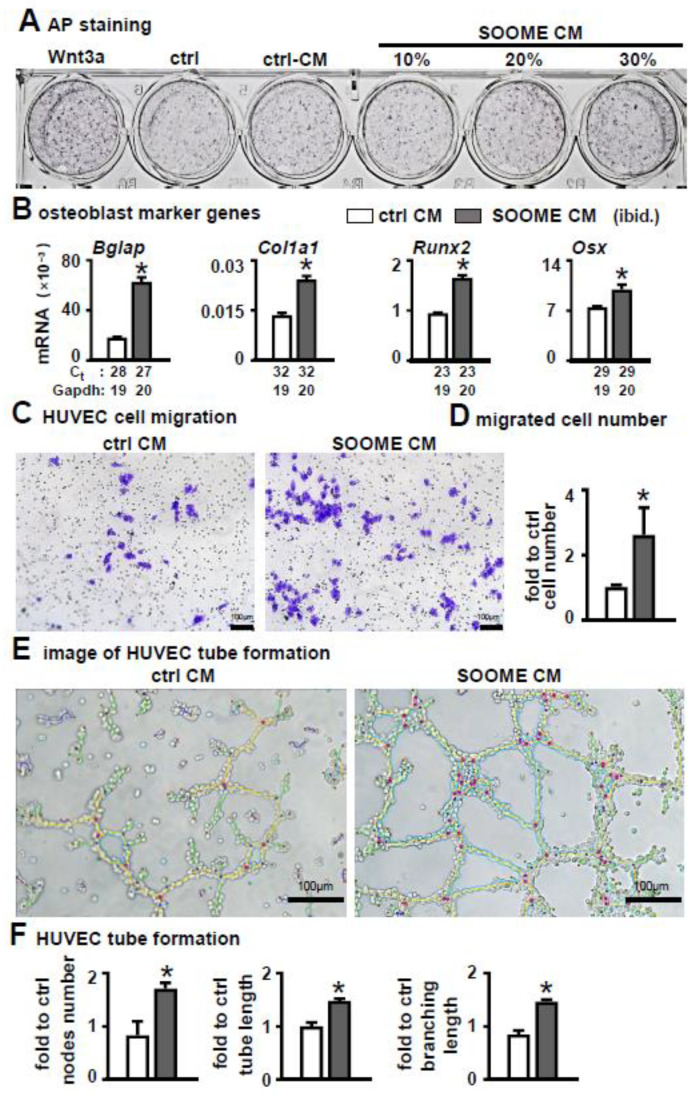
Effects of conditioned medium of SOOME on osteoblast differentiation and angiogenesis. The conditioned medium was collected from the supernatant of the medium of 24 h culture of SOOME and control CM. (**A**,**B**) Effect of conditioned medium on osteoblast differentiation of ST2. Osteoblast differentiation assays for (**A**) AP staining and (**B**) qPCR detection of the expression of osteoblast marker genes with or without conditioned medium. (**C**–**F**) Effects of conditioned medium on angiogenesis. Endothelial cell migration assay: imaging (**C**) and quantification (**D**) of migrated cells cultured in the transwell chamber with conditioned medium for 48 h; Imaging (**E**) and quantification of and node formation (**F**) of HUVEC cells cultured on Matrigel with conditioned medium. The * symbol indicates *p* < 0.05 versus control conditioned medium by *t*-test; n = 3. Scale bar = 100 µm. Green = branches; orange = master segments; blue sky = meshes; red surrounded by blue = nodes; Ct, cycle number detected by qPCR machine for a relative amount of target genes.

To investigate whether the conditioned medium from SOOME had similar function to SOOME, we tested it on osteoblast differentiation and angiogenesis. The results demonstrated that the conditioned medium was able to increase the differentiation of ST2, which was proven by AP staining and the expression of osteoblast marker genes (Figure 7A,B). 

Additionally, the conditioned medium promoted the migration of HUVEC cells (Figure 7C). The number of migrated cells increased 3-fold versus the control group (Figure 7D). Moreover, the conditioned medium increased tube formation and the number of nodes: total length of tubules and branching were increased by 2.1- and 1.6-fold in the conditioned medium of the SOOME compared to DMSO group (Figure 7E,F).

## 4. Discussion

The reconstruction of an osteogenic microenvironment of natural bone tissue is the goal of basic research on bone tissue regeneration (BTE). We originally found that activating osteocytic Wnt signaling promotes bone formation and bone resorption in mice, and considered that Wnt signaling-activated osteocytes may be an osteogenic microenvironment that may be potentially applied in BTE. We found that small molecule S24 activates canonical Wnt signaling in the MLO-Y4 osteocytic cell line. Interestingly, after S24 treatment of MLO-Y4 for 24 h, Wnt signaling can be maintained for three days,—a time sufficient to induce differentiation of bone marrow stromal cell ST2 to osteoblasts and mineralize as well. These data suggest that S24-treated osteocytes are an osteogenic microenvironment (SOOME), which can be proven by the converse inhibition of Wnt inhibitor triptonide. Moreover, we have verified the function of SOOME in 3D bioprinted modules by alternatively printing hard material strips along with cell-laden hydrogel strips. In doing this, we demonstrated that SOOME stably supports the growth and development of ST2 cells with a 7-day cell survival rate as high as 91.6%, and linearly increases proliferation activity. Therefore, SOOME promotes osteogenic differentiation and mineralization for 28 days. In addition, SOOME can recruit endothelial cells for angiogenesis, which has potential value in the application of bone tissue engineering.

The biggest innovation of this study was to find a small molecule, instead of gene operation, to activate Wnt signaling in osteocytes. After removal of the small molecule, the osteocytes still had strong biological activities, such as osteogenesis and angiogenesis. In fact, after 3D printing technology solves the problem of material shape matching, the next bottleneck will be how to improve the biological activities of materials for modern BTE, as this controls the development of BTE. However, as reported in 2016, an integrated printing of hard materials and stem cell-laden hydrogel has been invented to reconstruct a module with hard material for mechanical support and cells for bioactivity [47]. Inducing a module to vascularize bone in vivo after implantation will be a milestone in the field, pointing to the future 3D approach for clinic usage as reviewed [48,49]. In the time since those publications, however, such research has made little progress. For example, innovative materials, such as inorganic salts, borates, silicates or magnesium, release active ions with minimum bioactivity [44], and even cell-cell interactions, such as MSC and HUVEC co-culture, induce osteogenic and angiogenic development [15,50]; however, the capacities for cell survival rate and osteogenic differentiation and mineralization in vitro need to be improved. SOOME prepared in this study not only has higher safety, but also can induce the differentiation and mineralization of target cells in vitro for up to 28 days. This is of great advantage for osteogenesis and angiogenesis. 

This study found and prepared the osteogenic microenvironment SOOME. The above characteristics of SOOME show that it can be applied in 3D modules for translational medicine. Other scientists have also shown or reported that osteocytes are target cells that regulate osteoblast development [44,51,52]. These results suggest that osteocytes, as the main cell type controlling the bone microenvironment, mediate bone anabolism. However, the molecular mechanism of osteocytes in bone regeneration remains to be elucidated. We measured that SOOME upregulated the expression of PGE2 receptor *EP4* both by itself and when in co-culture with ST2 cells (Figure S3D,E), and we found that the Wnt signal inhibitor triptonide could completely inhibit the osteogenic differentiation in the co-culture (Figure 3A–C). These results suggest that SOOME produces *PGE2* or Wnt ligands to promote osteogenic differentiation of ST2 cells.

Application of SOOME on osteogenesis and angiogenesis is very safe, with no possibility that small molecules induce unbridled activation of Wnt signaling of other cells or tissues.

Unlike other Wnt agonists, S24 does not directly inhibit the activity of GSK3β. Because GSK3β is also involved in many other signal transduction pathways, directly inhibiting the activity of GSK3β may cause side effects or other pathological functions [31]. In addition, the outcome of the SOOME 3D modules was somewhat unexpected because S24-activated osteocytic Wnt signaling lasted for three days and no more than five days (Figure 1D), whereas the culture time of the 3D scaffolds was up to 7–28 days, indicating that the osteocytes with 3-day activation of Wnt signaling are sufficient to initiate osteogenic differentiation; they are probably at the stage for cell fate determination and differentiation as a spatial-temporal regulation in development. Previous studies have shown an increase of bone mass by direct oral administration or in vivo injection of Wnt activators, however, these drug delivery methods activated systemic Wnt signaling and are prone to induce unknown systemic side effects [51,52,53]. Some studies have attempted to deliver drugs with hydrogel, but uncontrollable release and locally high concentration of the drugs may lead to cell damage, resulting in safety concerns [54]. In this study, we treated osteocytes with drugs for one day then withdrew the drugs. The resultant SOOME promoted osteogenic differentiation of ST2 cells in the absence of S24. In particular, SOOME activated Wnt signaling for only five days, which could reduce unexpected side effects of direct action drugs or overactivation of cell signal pathways. One of the highlights of this study is that the reconstructed SOOME was free of drugs, which means it would not affect other cells or tissues around, making it safe for bone tissue engineering.

In addition, SOOME may induce angiogenesis of endothelial cells. Previous studies reported that canonical and noncanonical Wnt signaling promote angiogenesis by affecting the proliferation and migration of endothelial cells [55]. Blood vessels play an important role in the process of bone formation. They are not only nutrient delivery organs for nutrient diffusion, cell proliferation and new bone tissue growth, but also play a key role in regulating cells and signal molecules involved in bone regeneration [56,57]. Therefore, this part of the discovery further demonstrates the prospects for application of scaffolds containing SOOME to realize osteoblast function.

Although we found that the SOOME has the function of osteogenic differentiation on ST2 cells and angiogenesis on endothelial cells, it is one-sided to define S24-activated MLO-Y4 as an osteogenic microenvironment. In order to determine whether the SOOME has other osteogenic factors, we performed follow-up studies to analyze the influence of supernatant secreted from the SOOME on ST2 cells and endothelial cells. The results indicated that the supernatant could promote osteogenic differentiation of ST2 and migration and angiogenesis of endothelial cells (Figure 7). The results support that Wnt-activated osteocytes perform the above functions not only through cell-cell communication, but also partially by paracrine biological factors. This result also verifies that the postulation of the osteogenic microenvironment we reconstructed is successful even by unknown mechanisms. This will also be our next area of focus for research. 

## 5. Conclusions

The osteocyte with SKL2001-activated canonical Wnt signaling is a safe osteogenic microenvironment. Both the SOOME itself and its cell-free culture supernatant improve biological activities, such as cell survival, proliferation, differentiation towards osteoblasts, mineralization and angiogenesis. We further verified the biological activity of SOOME in a 3D model to a good proximation of the real in vivo microenvironment, which is valuable for bone tissue regeneration and repair.

## Figures and Tables

**Table 1:** Sequences of primers used for RT-PCR (mouse).

Primer	Forward	Reverse
*Gapdh*	GCACAGTCAAGGCCGAGAAT	GCCTTCTCCATGGTGGTGAA
*beta-actin*	AGAGGGAAATCGTGCGTGAC	CCATACCCAAGAAGGAAGGCT
*Lef1*	TACCCCAGCCAGTGTCAACA	TCCATGATAGGCTTTGATGACTTTC
*Axin2*	TGCAGGAGGCGGTACAGTTC	GCTGGAAGTGGTAAAGCAGCTT
*Bmp4*	GAGGAGTTTCCATCACGAAGA	GCTCTGCCGAGGAGATCA
*Smad6*	AAGATGCTGAAGCCGTTGGT	CGAACTCCAGTATCTCCGCTTT
*Alpl*	CACGGCGTCCATGAGCAGAAC	CAGGCACAGTGGTCAAGGTTGG
*Runx2*	CCGGTCTCCTTCCAGGAT	GGGAACTGCTGTGGCTTC
*Osx*	CCCTTCTCAAGCACCAATGG	AAGGGTGGGTAGTCA TTTGCA TA
*Bglap*	CAGCGGCCCTGAGTCTGA	GCCGGAGTCTGTTCACTACCTTA
*Col1a1*	GACAGGCGAACAAGGTGACAGAG	CAGGAGAACCAGGAGAACCAGGAG
*Ibsp*	CAGAGGAGGCAAGCGTCACT	GCTGTCTGGGTGCCAACACT
*Ang-1*	ACTAGTAGTACAATGACAGTTTTCCTTTCC	AGATCTTCAAAAGTCCAAGGGCCGGATCAT
*EP2*	GATGAAGCAACCAGAGCAGAC	CAGAGAGGACTCCCACATGAA
*EP4*	GCCCTCTCCTGCCAATATAAC	TTTCAACACTTTGGCCTGAAC

## Data Availability

The data presenting in this study are available on request from the corresponding author.

## Supplementary Materials

The following are available online at https://www.mdpi.com/article/10.3390/cells11050831/s1. Figure S1: SKL2001-stimulated osteocytes promote osteoblast differentiation of ST2 cells in a dose-dependent way; Figure S2: Different effects of S24-treated NIH 3T3 fibroblasts and MLO-Y4 osteocytes on osteoblast differentiation of bone marrow stromal cell ST2; Figure S3: Investigation of potential factor(s) of SOOME on osteoblast differentiation; Figure S4: MLO-Y4 cells treated with S24 hardly change the expression of marker genes of osteoblasts and osteocytes; Figure S5: ST2 cells in the hydrogels are much less differentiated into osteoblasts without MLO-Y4.
