# Supplementary material for: The Osteocyte Stimulated by Wnt Agonist SKL2001 Is a Safe Osteogenic Niche Improving Bioactivities in a Polycaprolactone and Cell Integrated 3D Module
Source: Cells. 2022 Feb 28;11(5):831. doi: 10.3390/cells11050831 (PMC8909416; doi:10.3390/cells11050831)
Supplement: Supplementary file 1 [file cells-11-00831-s001.zip › cells-1519717-supplementary.pdf]

Supplementary data

Title: The osteocyte stimulated by Wnt agonist SKL2001 is a safe osteogenic niche improving bioactivities in a polycaprolactone and cell integrated 3D module

s1: SKL2001-stimulated osteocytes promote osteoblast differentiation of ST2 cells in a dose-dependent way.

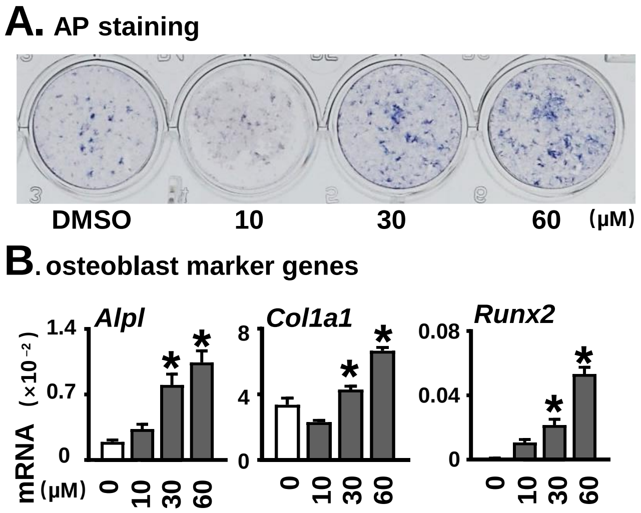

Figure S1. Effects of SKL2001 amount in the treatment of osteocytic MLO-Y4 on osteoblast differentiation of mouse stromal cell ST2. S24 treated MLO-Y4 cells at concentrations as indicated for 24 hours, and then S24 was washed away. ST2 cells were co-cultured with these treated MLO-Y4 for 3 days and were tested for osteoblast differentiation by AP staining (**A**) and the expression osteoblast marker genes (**B**). * p<0.05 versus DMSO control by One Way Anova, n=3. Ct, cycle number detected by qPCR machine for a relative amount assay for relative amount of target genes.

s2: Different effects of S24-treated NIH 3T3 fibroblasts and MLO-Y4 osteocytes on osteoblast differentiation of bone marrow stromal cell ST2

Materials and Methods:

NIH 3T3 cells were purchased from Yuchi Biology (Shanghai, China). Cells were cultured in DMEM (Invitrogen, USA) supplemented with 10% fetal bovine serum, 1% glutamine, 1% penicillin/streptomycin, and 1% gentamicin [1].

AP staining was performed to measure osteoblast differentiation in the co-cultures of S24-treated NIH 3T3 or MLO-Y4 for 24 hours, and then co-cultured with ST2 or ST2/NIH 3T3 cells, respectively for 3 days.

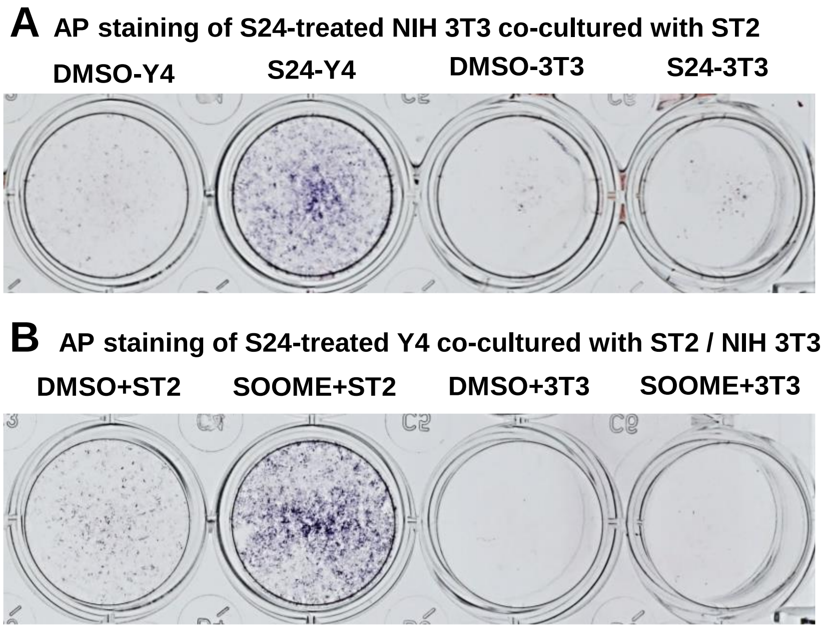

Figure S2. Different effects of S24-treated NIH 3T3 / MLO-Y4 on osteoblast differentiation of bone marrow stromal cell ST2. NIH 3T3 (**A**) or MLO-Y4 (**B**) was treated with 60μm S24 for 24 hours, and then co-cultured with ST2 or NIH 3T3, respectively for 3 days. Y4, MLO-Y4; 3T3, NIH 3T3. DMSO, DMSO-treated MLO-Y4; SOOME, S24-treated MLO-Y4.

[1] W. Rao, G. Xie, Y. Zhang, S. Wang, Y. Wang, H. Zhang, F. Song, R. Zhang, Q. Yin, L. Shen, H. Ge, OVA66, a tumor associated protein, induces oncogenic transformation of NIH3T3 cells, PLoS One 9 (2014) e85705-e85705. 10.1371/journal.pone.0085705.

s3: *Investigation of potential factor(s) of SOOME on osteoblast differentiation.*

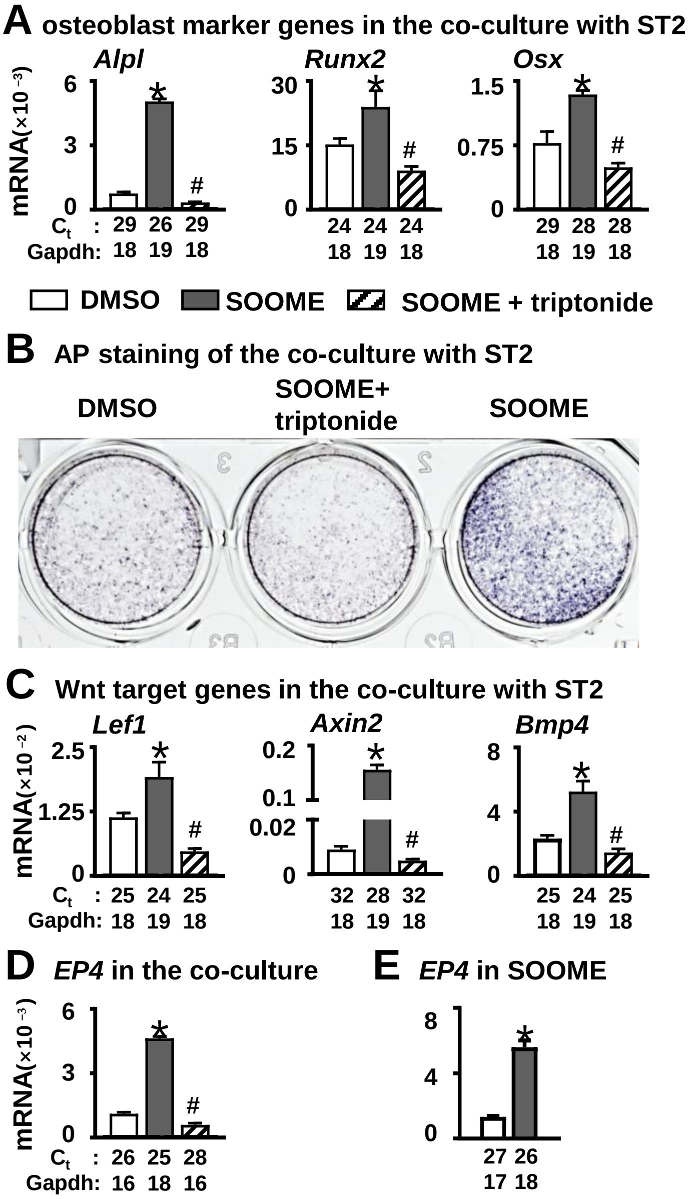

Figure S3. Effects of potential osteogenic factor(s) from SOOME on osteoblast differentiation of ST2 cells. The co-culture system of SOOME and ST2 was treated with Wnt inhibitor triptonide (10nM) for 3 days. Assays of osteoblast differentiation were performed by (**A**) qPCR detection of the expression of osteoblast marker genes and (**B**) AP staining; qPCR detection of the expression of Wnt target genes (**C**) and EP4 (a PGE2 receptor) in the co-culture system (**D**) or in SOOME (E). **p* < 0.05 versus DMSO control, # *p*<0.05 versus SOOME, by One Way Anova, n=3. Ct, cycle number detected by qPCR machine for a relative amount of target genes.

s4: MLO-Y4 cells treated with S24 hardly change the expression of marker genes of osteoblasts and osteocytes.

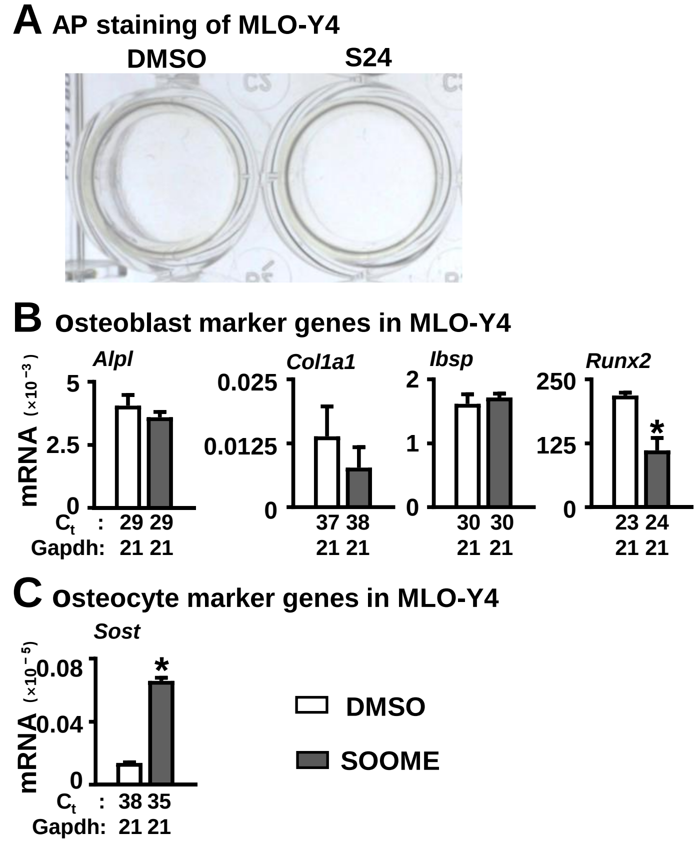

Figure S4. Detection of MLO-Y4 and SOOME on the expression of osteoblastic and osteocytic marker genes. (**A**) AP staining; qPCR detection of the expression of marker genes of osteoblast's (**B**) and osteocyte marker genes (**C**) in MLO-Y4. * $p < 0.05$ versus DMSO control, by t-test, n=3.

s5: ST2 cells in the hydrogels are much less differentiated into osteoblasts without MLO-Y4

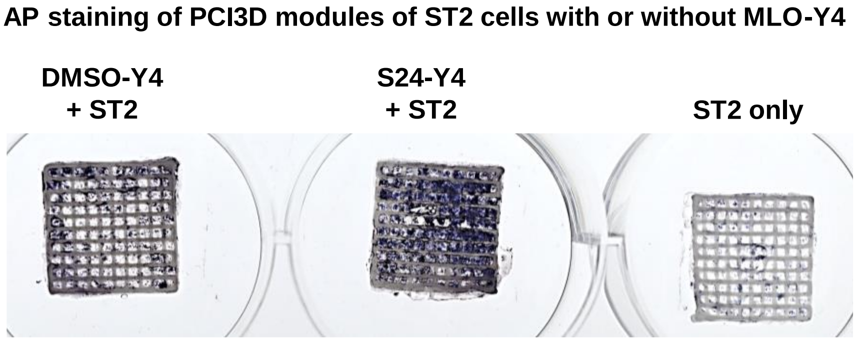

Figure S5. Hydrogel has less effect on osteoblast differentiation of ST2 cells without treated or untreated MLO-Y4 cells. AP staining was performed on PCI3D printed modules of PCL bundle and cell-laden hydrogel. Bone marrow stromal cell line ST2 was printed with or without MLO-Y4 at 4:1 cell ratio for 7-day co-culture. MLO-Y4 was treated with 60 μM S24 or DMSO (control) for 24 hours before 3D bioprinting. Y4, MLO-Y4; S24, SKL2001.
